# The Resilience and Resistance of an Ecosystem to a Collapse of Diversity

**DOI:** 10.1371/journal.pone.0046135

**Published:** 2012-09-27

**Authors:** Andrea S. Downing, Egbert H. van Nes, Wolf M. Mooij, Marten Scheffer

**Affiliations:** 1 Aquatic Ecology and Water Quality Management Group, Department of Environmental Sciences, Wageningen University, Wageningen, The Netherlands; 2 Department of Aquatic Ecology, Netherlands Institute of Ecology, Wageningen, The Netherlands; University of Southampton, United Kingdom

## Abstract

Diversity is expected to increase the resilience of ecosystems. Nevertheless, highly diverse ecosystems have collapsed, as did Lake Victoria’s ecosystem of cichlids or Caribbean coral reefs. We try to gain insight to this paradox, by analyzing a simple model of a diverse community where each competing species inflicts a small mortality pressure on an introduced predator. High diversity strengthens this feedback and prevents invasion of the introduced predator. After a gradual loss of native species, the introduced predator can escape control and the system collapses into a contrasting, invaded, low-diversity state. Importantly, we find that a diverse system that has high complementarity gains in resilience, whereas a diverse system with high functional redundancy gains in resistance. Loss of resilience can display early-warning signals of a collapse, but loss of resistance not. Our results emphasize the need for multiple approaches to studying the functioning of ecosystems, as managing an ecosystem requires understanding not only the threats it is vulnerable to but also pressures it appears resistant to.

## Introduction

Diverse systems are generally considered more constant [1], reliable [2], predictable [3], and less prone to change [4] or invasion [5] than simple systems. In spite of this, diverse systems have been known to suddenly collapse: from the global scale prehistoric mass extinctions [6] to the smaller scale recent cases of Caribbean coral reefs [7], and of Lake Victoria’s cichlid diversity [8].

Biodiversity is known to benefit systems through several mechanisms. Ecosystem experiments reveal that species-rich systems can exploit resources more efficiently than species-poor systems [9], [10]. This mechanism is known as complementarity and illustrates how systems that contain a high diversity of species can reach a higher biomass. Also, a large number of species can imply some level of functional redundancy: the loss of one species has a smaller effect in a diverse system than in a species-poor one. This is the insurance effect [1], [11]. High species diversity also implies higher chances of having species that efficiently fulfill functions presented by their environment – which is known as the sampling (or selection) effect. Furthermore, it is often argued that for co-existence to be possible, even functionally redundant species need to differ in some aspects, including in their susceptibility to threats or to changes to their system (response diversity) [12], [13]. Response diversity, sampling and insurance effects are said to increase the resilience and resistance of an ecosystem.

Though it seems clear from experiments and theoretical work that diversity has effects on the stability of an ecosystem in the broad sense – and is the subject of a sixty year-old debate known as the diversity-stability debate [5], [14], [15], diversity has seldom been explicitly connected to ecosystem resilience and resistance. We here describe the resilience of a system by the size of its basin of attraction in the stability landscape [16], which can also be thought of as the maximum perturbation a system can withstand and remain in the same state [17]. Resistance also represents the amount of perturbation that a system can withstand and remain in the same state, but it is not associated to a change in the size of basin of attraction, rather to a change in persistence, or inertia, of a system state [18]. While it has been suggested that diversity loss can cause loss of system resilience by reducing the size of the basin of attraction of an ecosystem state [19], mechanisms associating diversity to critical transitions have not yet been identified.

Critical transitions – such as those that shape the dynamics of a shallow lake shifting between its clear and turbid states [20] – occur when environmental conditions change and reshape a basin of attraction. This can reduce a system’s resilience until it easily and rapidly slips into an alternative state – or basin of attraction. A sudden system collapse can also happen when a perturbation knocks a system out of its basin of attraction into an alternative one [17], [21]. Critical transitions and alternative attractors require the presence of positive feedbacks: under a weak feedback, the system can react smoothly to environmental changes. However, a strong enough positive feedback can yield alternative stable states, in which case a system can exist in different states for the same range of environmental conditions [17]. The presence of alternative attractors in systems has important management consequences because they imply hysteresis, whereby the shift from one state (basin of attraction) to the next does not occur under the same conditions as the reverse shift back to the initial state [17].

Diverse ecosystems have been associated with critical transitions, yet the feedbacks behind these transitions have not yet been linked to diversity, but rather to keystone species [17], [22], [23]. The idea that diverse communities as a whole exert a feedback on their environment or on new-coming species is however not new [24]. In coral reefs for example, several trophic groups of grazing fish are necessary to suppress the growth of macro-algae, if grazing is insufficient, the system shifts from a coral-dominated state to one where macro-algae dominate [25]. A diversity-emergent feedback might also be associated to the story of Lake Victoria’s diversity collapse. In this case, more than 300 species of haplochromine cichlids that used to occupy every trophic level of Lake Victoria’s system suddenly disappeared and were replaced by the introduced Nile perch [8] – though only 30 years after the introduction of Nile perch. One hypothesis for this delayed and sudden shift is that native cichlids might have initially controlled their introduced predator by predating on Nile perch eggs, but that increased eutrophication and fishing caused a slow decline in cichlid diversity. This gradual diversity loss eroded the resilience of the system until the egg-predation control mechanism failed, allowing Nile perch to suddenly boom [26]. Inspired by this hypothesis, we investigate – using a simple multi-species model – how a strong positive feedback can emerge from a diverse system and how it affects the mechanisms that confer resilience to diverse systems, looking for insight into the paradox of diversity collapses. We here primarily aim to investigate the role of diversity itself in shaping the resilience of systems when an inconspicuous diversity-emergent feedback is at play and to understand some of the implications of diversity loss for a system in the presence of such a feedback.

## Results

**Figure 1:**
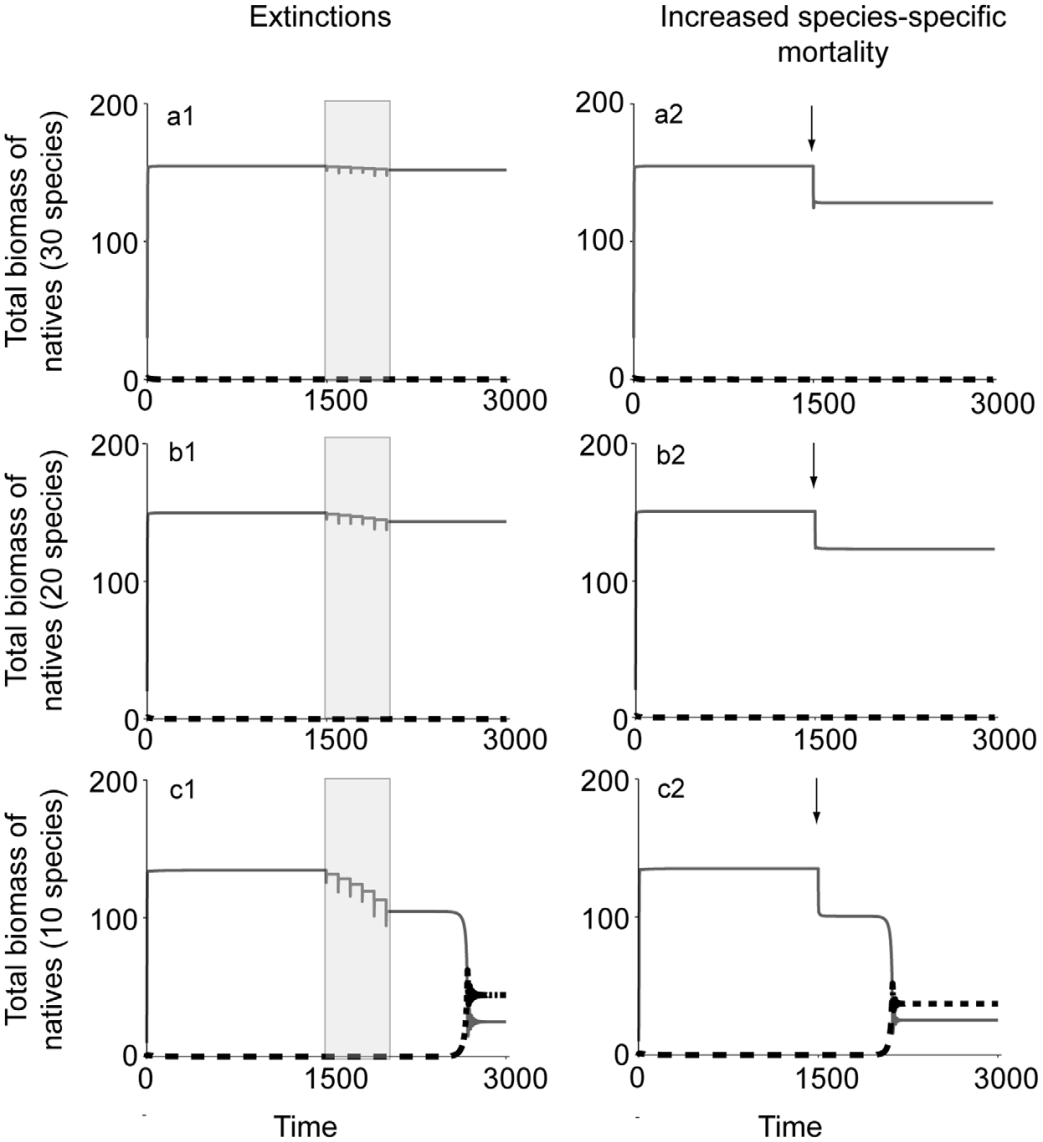
The effects of diversity and diversity loss on the outcome of the introduction of a predator into a diverse native community. Initially, native species prevent the introduced predator from invading by reducing the predator to a low biomass. After species extinctions (shaded areas) or an increase in species-specific mortality m_F,I_ (arrow), at low diversity, the feedback mechanism fails and the introduced invades very suddenly. Low diversity communities have a lower initial biomass and the effect of diversity loss has a larger effect on the total biomass of less diverse systems – see supporting information S2. For clarity and ease of comparison between simulations, we here use a fixed rather than random interspecific competition coefficient (*α_i,j_*) (p = 0.0015; e = 0.6; r = 1; g = 0.7; H = 20; m = 0.22; *α_i,j_*_ = _0.3; K_i = _50, m_F,i_ = [0,0.5], I = 5).

In our model, a high number of native species can effectively suppress the introduced predator to a very low biomass (fig. 1). However, following species extinctions or an increase in species-specific mortality, a less diverse system can undergo a catastrophic collapse and shift to a state where the introduced predator has invaded and dominates (fig. 1c1 and 1c2, see supporting information S1 for results obtained using functional response type II). When running the model with consecutive native species’ extinctions, we see that at high diversity, species’ extinction usually only leads to a slight decrease in the total biomass of the native community (fig. 1a1, 1b1), but when starting from a lower initial diversity a few consecutive species extinctions causes a relatively large biomass loss that ultimately leads to collapse (fig. 1c1).

At low diversity, the results stemming from a random choice of competition coefficients are more variable than at high diversity. Therefore, for clarity and easier comparison of different diversity treatments in figure 1, we use a fixed value for interspecific competition (*α_i,j_*) – representing a community average. When we use a random range of competition coefficients within the native community, the predator invasion causes the native system to collapse to a state of further reduced diversity.

**Figure 2:**
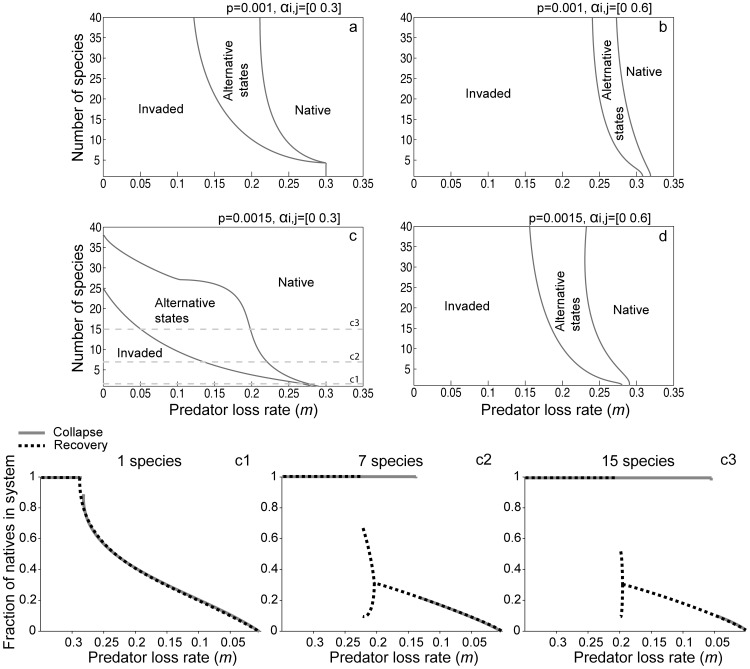
Effect of diversity and predator loss rates on dynamics. Stronger competition decreases the range of predator loss rates for which there are alternative stable states (b and d, versus a and c). In (c) with 25–38 native species, the predator only dominates if it is already dominant, it cannot invade a native-dominated system. To make a), b), c) and d), we ran the model in two sets of 20 runs for each number of species, starting in a native state and decreasing the predator loss rate (*m*); then starting from the invaded state and increasing *m*. Lines are averages of 20 runs. c1), c2) and c3) represent cross sections of c) at 1, 7 or 15 species: following equilibria as the system shifts from native to invaded and back. (e = 0.6; r = 1; g = 0.7; H = 20; m = 0.2; K_i_ = 50).

We use two-dimensional bifurcation analyses to systematically check the effect of species diversity on the position of the critical transitions for different parameter settings (fig. 2). As diversity increases, the range of conditions over which the system has alternative attractors also increases. Increased diversity thus makes the system more resilient to invasion by pushing the threshold to collapse away, but once a diverse system has collapsed, it is also further away from the conditions necessary for recovery. This effect is limited when competition coefficients (*α_i,j_*) are higher (fig. 2b and 2d), but it is exacerbated by both low competition and high feedback rate (*p*_i,_) (fig. 2c). The predator is more easily suppressed when competition between native species is lower, i.e. there is a larger range of parameter conditions under which the system is in a native-only state for low competition values (fig. 2a and 2c).

In our simplified keystone-species model, one productive species can resist invasion by the introduced predator (supporting information S2), but a diverse community can achieve invasion resistance with lower species-specific carrying capacities and weaker feedback rates.

**Figure 3:**
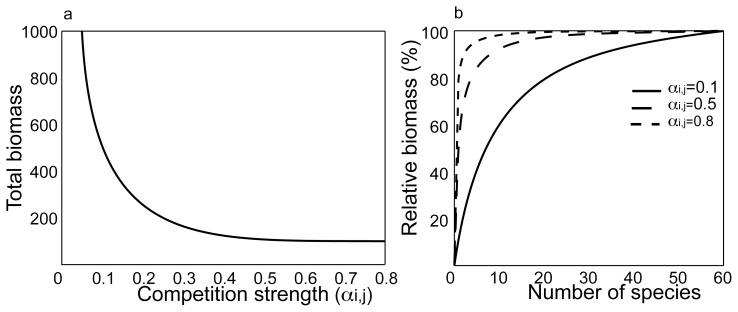
Biomass-diversity relationship. When interspecific competition is lower than intraspecific competition several species can make up more biomass than a single species on a given amount of resource. The total biomass made up by 100 species decreases with increasing interspecific competition strength (left hand panel). Here it is complementarity that increases the productivity in the system. When competition is stronger (high *α_i,j_*), a few species quickly make up the total biomass (right hand panel). This also illustrates how when competition is high, the decrease in biomass with diversity loss happens more suddenly and at a lower diversity (dashed lines).

The way we model our diverse native community – where interspecific competition is lower than intraspecific competition – implies a certain level of complementarity between species. Weak competition reflects high niche complementarity, and translates into more efficient use of resources and thus higher productivity (fig. 3a). Therefore, our diverse communities make up a higher biomass than species-poor communities (c.f. fig. 1). When competition is strong (*α_i,j_* closer to 1), fewer species bring the total biomass of the community to its maximum, which also implies that a diversity decline in a highly competing community leads to a more abrupt loss of biomass than in a more complementary community (fig. 3b, dashed lines).

The number of extinctions necessary to cause a collapse is thus a complex function of the number of species present in the native community, the average strength of intra-specific competition within this community and of the negative feedback exerted by the native species, the variability in competition and feedback – especially at low diversity – and on the predation rate of the introduced predator.

**Table 1:** Model parameters.

Parameter	Name	Dimensions
*g*	Predator foraging rate	Time^−1^
*H*	Feeding half saturation	Biomass
*r*	Native per capita growth rate	Time^−1^
*α_i,j_*	Competition coefficient	
*K_i_*	Prey carrying capacity	Biomass
*e*	Conversion efficiency	
*m*	Predator background mortality rate	Time^−1^
*p_i_*	Predator mortality caused by prey *i*	Time^−1^ Biomass^−1^
*m_F, i_*	Extra mortality rate on native species *i*	Time^−1^

## Discussion

We here show with a very simple model how a community-wide feedback can make a diverse community more resilient to invasion than a species-poor system and how this feedback might imply that the loss of a few species could lead to a critical transition. The mechanism through which a diverse community acquires this resilience – here through increased productivity – is relevant for many other ecosystems that have alternative states, for instance systems that can switch to an overgrazed state [27].

The small feedback mechanism we model could indeed stand as a possible explanation to Lake Victoria’s mystery: the collapse of most cichlid species happened within a few years, but only thirty years after the introduction of Nile perch. The diversity collapse and Nile perch boom followed long-term increases in fishing pressure and eutrophication, two processes that had negative impacts on native species [28]. Interestingly, the collapse of haplochromine cichlids in Lake Victoria was accompanied by an increase in the abundance of the shrimp *Caridina nilotica*, a competitor of the largest cichlid trophic groups [29]. This shrimp became an important food source for Nile perch, but is an unlikely threat to juvenile Nile perch: it could thus represent a case where the insurance effect – seen in shrimp replacing vanishing haplochromines – decreased the resilience of the community to invasion by providing more food for Nile perch but without negatively affecting Nile perch recruitment. However, after the disappearance of their haplochromine prey, Nile perch cannibalized their own young more [30]. If the feedback mechanism we propose had any role in the Nile perch invasion, it would be important to know to what extent and under what conditions cannibalism by Nile perch compares to egg-predation by haplochromines. Indeed, these two processes could play an important role in determining the resilience of the current invaded-state of Lake Victoria to Nile perch fishing [30], [31].

Caribbean coral reefs show similar albeit more complex diversity collapses that can also be compared to the mechanism we model. The native diverse state comprised high coral cover and a diversity of grazing fish. With fishing and eutrophication, fish stocks and coral cover declined, and a single species of urchins took over the task of grazing on macro-algae. This simplified system rapidly collapsed leaving a fully barren state [7]. One of the main feedbacks maintaining the coral state is grazing pressure by a diverse assemblage of grazing herbivorous fish that use corals as a habitat [25]. Additionally, a recent study by Price et al. (2011) [32] demonstrates how coral diversity promotes functional diversity in fish: this could represent another important feedback mechanism that might greatly increase a reef’s resilience but that could also set it up for a catastrophic shift should a minimum diversity threshold be crossed.

The effect of size-specific mortality and of ontogeny – two processes that are implied but not modeled in the present study – have been shown to influence the co-existence of predators and their prey and cause regime shifts between alternative stable-states [33], [34]. Interestingly, our findings represent an up-scaling of that seen in culling experiments on a fish population, where size specific-mortality could lead to the compensatory growth of different size-classes [35]. These processes have also recently been studied in fuller food web experiments that include a larger number of species and where the introduction of a predator results in shifts to alternative stable-states [36]. We suggest an interesting follow-up on this research could lie in explicitly investigating the combined effects of diversity and ontogenetic or size-specific interactions and mortality, to evaluate whether they have reinforcing, neutral or cancelling effects on the resilience and stability of a system.

An essential ingredient of our model is the productivity-diversity relationship, where it is hypothesized that a productive system promotes diversity to a certain extent, and that diversity produces a higher yield (productivity) than a species-poor system [37]. This relationship has been observed in many experiments and systems [1], [12], [38]. In our model, diversity leads to higher productivity through complementarity, as we assume lower inter- than intraspecific competition values. In real systems, however, this relationship can also be driven by other mechanisms: through resilience to microbes [39], functional diversity [11], [40] or through niche partitioning [9]. Our findings suggest that these processes could reinforce the effects here produced with complementarity, and perhaps even suffice to shape resilience in the presence of a small feedback, such as the one we model.

Despite its simplicity, our model allows us to differentiate resilience obtained from the diversity-productivity relationship to resistance acquired from the insurance effect. Because the collapse threshold is here a biomass-limit, productivity makes the system more resilient to an invasion by increasing its distance from the point of collapse (fig. 3). In contrast, the insurance effect reduces the effect of each species’ extinction on the total biomass of the native community (fig. 3b) and thus increases the system’s inertia – or resistance. Resilience and resistance each seem to have their own trade-off: the ability to ward-off invasion that comes with increased productivity – resilience – also comes at the cost of increasing the range of conditions for which there are alternative attractors. The insurance that comes with functional redundancy – resistance, even though it does not lead to hysteresis on its own, does not in itself give the ability to withstand invasion. In this perspective, Caribbean reefs seem to display more of a resistance effect: low diversity stands of grazing urchin initially succeeded in preventing algal growth – the loss of species did not initially reduce the critical biomass necessary to control the invading algae. The resilience of this urchin-dominated community was however already compromised, as pathogens wiped out this low-diversity system and critically reduced its productivity. Perhaps this illustrates a mechanism similar to that seen by Schnitzer et al. (2011), whereby diversity in grasslands increased the resilience of plants to disease and lead to a strong diversity-productivity relationship.

This distinction between resistance acquired through the insurance mechanism and resilience obtained through the diversity-productivity relationship is relevant when identifying the causes of diversity decline. Habitat destruction, such as forest clearing, might for example kill different species indiscriminately across system functions, whereas eutrophication or climate change might primarily affect species carrying out a single function – possibly species that compete quite strongly. Our study suggests that these different threats, though they might have the same impact on the number of species present, might have very different effects on ecosystem resilience and on the reversibility of a collapse.

The difference in how a system becomes more vulnerable – be it through a decrease in the system’s inertia (resistance) or through changing the size of the system’s basin of attraction (resilience) – will probably also affect the foreseeability of a critical transition. When loss of diversity implies a change in the basin of attraction, the system can present early warning symptoms of resilience loss: the rate at which it recovers from minor disturbances is lower, this is known as critical slowing down. A system that looses in resistance, however, is not expected to show any such symptoms [41].

The mechanism we model is very simple and general: all species are prey to the invader, and all contribute to prevent invasion. In reality, it is more likely species all have different effects – the shrimp in the Lake Victoria hypothesis we present could be a good example of this. Our results here show a clear-cut biomass threshold above which the system is resistant to invasion and above which both the insurance effect and increased productivity can further increase resilience of the system. However, this threshold becomes blurred in situations where species are not equally efficient at controlling the invader or equally susceptible to predation and where some species are competitively superior to others. The overall effects of species extinctions are then even less predictable.

Our model illustrates how a small, no-cost feedback inconspicuously applied by individual species can be amplified in a diverse community to have a huge impact: consequences of diversity loss are not necessarily linear and not only a function of the number of species that are lost, a fact that is of high relevance to ecosystem monitoring and management. In effect, we show that both the level of functional redundancy that characterizes a community and the way in which the community is disassembled – across or within functional groups – might play a role on the resilience of a system and on the reversibility of a collapse. It is also important to remember that a small feedback mechanism is invisible until it fails, and consequences of diversity loss will tend to be very unpredictable. These findings reinforce the view that the key to preventing unexpected ecosystem changes lies in managing the resilience of ecosystems, and that resilience management should focus on maintaining biodiversity [42].

Our results therefore emphasize the necessity to have a broad view on system processes and functioning, taking into account not only the pressures it is vulnerable to, but also the ones it appears resilient to. Such insight can be gathered not only from past collapses in other systems but also from understanding the mechanisms that structure communities and confer resilience to a system.

## Methods

### Model

To describe the native diverse community, we used a Lotka-Volterra competition model (eq. 1), in which native species (*N_i_*) differ from each other in their competition coefficient (*α*_i,j_), chosen randomly from a uniform distribution. We assumed interspecific competition to be lower than intraspecific competition (0< *α_i,j_* <1), allowing a diverse community to emerge. The diagonal elements of the competition matrix (*α_i,i_*) – that reflect intraspecific competition – are by definition equal to one. As an option, we inflict an extra species-specific and biomass-dependent mortality rate (*m_F,i_*) on native species (by default, *m_F,i_* = 0). This extra mortality might for example represent a fishing pressure to which different species are unequally vulnerable.

In this model, the introduced predator (*I*) (eq. 2) grows from feeding on all species of the native community with an attack rate *g*, following a sigmoidal (or Holling type III) functional response (eq. 1) with a half saturation value *H* and assimilation efficiency *e*. The sigmoidal functional response is commonly used for fish populations and assumes reduced predation at low prey densities. We tested the effects of this assumption by trying our model using a type II functional response (see supporting information S1 for results). It has been found by Guill (2009) [43] that the type of functional response does not influence the presence or absence of alternative stable states and critical transitions.

We assume that the predator has no food preference and feeds on each species proportionally to its biomass. The introduced predator has a loss rate (i.e. mortality and respiration) of *m*. In addition, each species of the native community causes additional mortality to the introduced predator that is proportional to its biomass (*p_i_ N_i_*) and has no cost or benefit to the native species. By default we assumed the feedback rate *p_i_* to be the same for all native species. Parameters are chosen so as to produce viable diverse communities; we test the effects of different parameter values in the model analysis (parameter descriptions given in table 1).


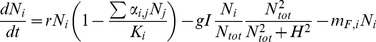
(1)


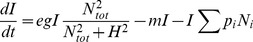
(2)

Where N_tot_ = Σ N_i_ and α_i,i_ = 1.

### Analysis

We explored the effects of different levels of diversity as well as of diversity loss through simulations of different scenarios. To test the effects of diversity on the outcome of the introduction of an invader, we first simply ran simulations with different numbers of native species (respectively 30, 20 or 10 species), no extra mortality on the native species (*m_F,i_ = *0), and the invader present from the start of the simulation.

To analyze the effects of diversity loss we applied two methods. In the one method, we tested the effects of sudden extinctions of individual species. For this we ran the simulations – again with different initial numbers of species – and set the biomass of a random native species to zero at chosen time steps. Our other method consisted in testing the effects of species-specific mortality rates within the native community. For this we ran the simulations, also starting with different initial numbers of species, but this time with the extra mortality (*m_F,i_* ).

To gain further insight into the effects of diversity on the feedback mechanism, we carried out numerical bifurcation analyses to identify system states for different parameter values, changing a control parameter incrementally and finding the equilibrium biomasses. We then tested the effects of diversity in two-dimensional bifurcation analyses by repeating the parameter analyses but with different numbers of species.

Simplifying the model to include only one strong keystone species, we conducted a more thorough model analysis (supporting information S2). We carried out phase plane analyses, identifying conditions under which populations do not change over time; we found system equilibria and analyzed their stability. Through our phase plane analyses we exposed the different possible system dynamics that our model yields. Then, in bifurcation analyses, we modified parameters two-by-two and delimited parameter spaces over which the different dynamics occur and determined how the system might change from one type of dynamic to the next (results of this analysis are in supporting information S2). All simulations were carried out with GRIND for MATLAB (http://www.aew.wur.nl/UK/GRIND) that solves differential equations with a Runga-Kutta method.

## Supporting Information

File S1 **Testing the functional response assumption.**
(DOC)

File S2 **Single native species model analysis.**
(DOC)
