# Supplementary material for: The Resilience and Resistance of an Ecosystem to a Collapse of Diversity
Source: PLoS One. 2012 Sep 27;7(9):e46135. doi: 10.1371/journal.pone.0046135 (PMC3459835; doi:10.1371/journal.pone.0046135)
Supplement: File S1 — Testing the functional response assumption. (DOC) [file pone.0046135.s001.doc]

**Resilience and resistance of an ecosystem to a collapse of diversity**

Andrea S. Downing, Egbert H. van Nes, Wolf M. Mooij, Marten Scheffer

**Supporting Information S1: Testing the functional response assumption**

To test the extent to which these results are driven by our assumption of a sigmoidal functional response, we ran the model using a Holling type II –monod-saturating type – functional response. The only difference observed using a type II functional response is that after the sudden collapse of native diversity, the system does not systematically stabilise into a stable invader-dominated state, but goes into predator-prey cycles. The basic mechanism we describe, whereby a decrease in diversity leads to a collapse, and that the threshold to collapse is set by a biomass threshold is robust to the change in functional response. The cycles seen here typically arise from an oversimplification of the predator-prey interaction: the monod-relation assumes equal feeding rate at low prey densities, and these cycles tend to disappear when one assumes not all prey are simultaneously equally vulnerable to the predator (e.g. in presence of spatial refugia for the prey).


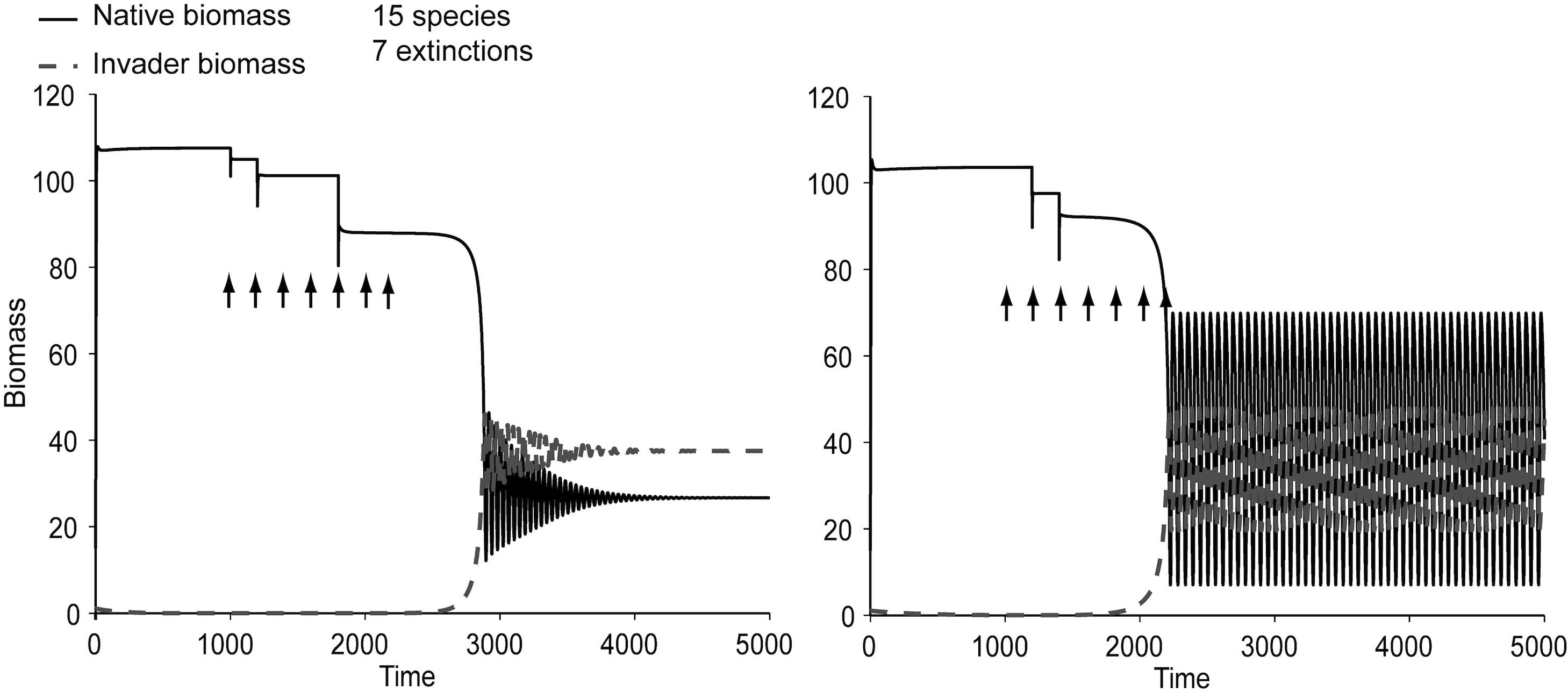


Figure S1: Invasion of the introduced predator when the predator-prey interaction is expressed with a Holling type II (monod) functional response. The mechanism we describe, following which the removal of native species eventually leads to a critical transition, is robust to the change in functional response. However, the monod functional response can lead to predator-prey cycles, thus making the results more complex to analyse, and overshadowing the basic mechanism we aim to describe. (p = 0.0015; e = 0.6; r = 1; g = 0.7; H = 20; m =0.2; α_i,j_ = [0 0,6]; K_i_ = 50). The results appear different for a same set of parameter conditions because of the random choice of competition coefficient α_i,j_.
