# Supplementary material for: The Resilience and Resistance of an Ecosystem to a Collapse of Diversity
Source: PLoS One. 2012 Sep 27;7(9):e46135. doi: 10.1371/journal.pone.0046135 (PMC3459835; doi:10.1371/journal.pone.0046135)
Supplement: File S2 — Single native species model analysis. (DOC) [file pone.0046135.s002.doc]

**Resilience and resistance of an ecosystem to a collapse of diversity**

Andrea S. Downing, Egbert H. van Nes, Wolf M. Mooij, Marten Scheffer

## Supporting Information S2: single native species model analysis

For easier analysis of the effects of different parameters, we simplified the model to include only one native species and the introduced invader. We used increased productivity – obtained with higher values of carrying capacity – as a proxy for increased diversity. Analysis reveals that there are four possible system dynamics, seen in phase planes featuring different configurations of nullclines, or lines of zero growth (fig. S2a-S2d). The native species’ nullcline is a curved line, where the native equilibrium is at highest biomass when invaders are absent, intersecting with the x-axis at carrying capacity, and at lowest biomass for high biomass of introduced predator. The introduced predator has two vertical nullclines representing native biomasses at which it can exist. The four configurations depicted in figure S2, with zero, one or two intersections between nullclines, are obtained by changing carrying capacity and predator loss parameters. We related each type of system dynamics to their area of the parameter space defined by control rate (*p*) and carrying capacity (*K*), illustrating how moving right along the x-axis (increasing *K*) causes the native isocline to stretch, and how moving up the y-axis (increasing *p*) causes the invader isoclines to merge. Intersections represent equilibria for both natives and the invader, the stability of these equilibria differs in the four configurations. At low carrying capacity there is not enough native-prey present to sustain an invader (fig. S2a), as carrying capacity increases, the native isocline intersects one invader isocline (fig. S2b): there is then enough native-prey for the predator, but the native species is not abundant enough to control the predator: the only system state is invaded. By increasing both *K* and *p*, the system enters alternative stable states parameter space (fig. S2c). Here initial conditions determine whether the predator or native species dominate. Increasing *K* or *p* further pushes the system through a Hopf bifurcation (fig. S2d), where the invaded state becomes unstable. Increasing control rate makes the two invader isoclines merge, predator losses are altogether too high and the only possible system state is the native one (fig. S2e).


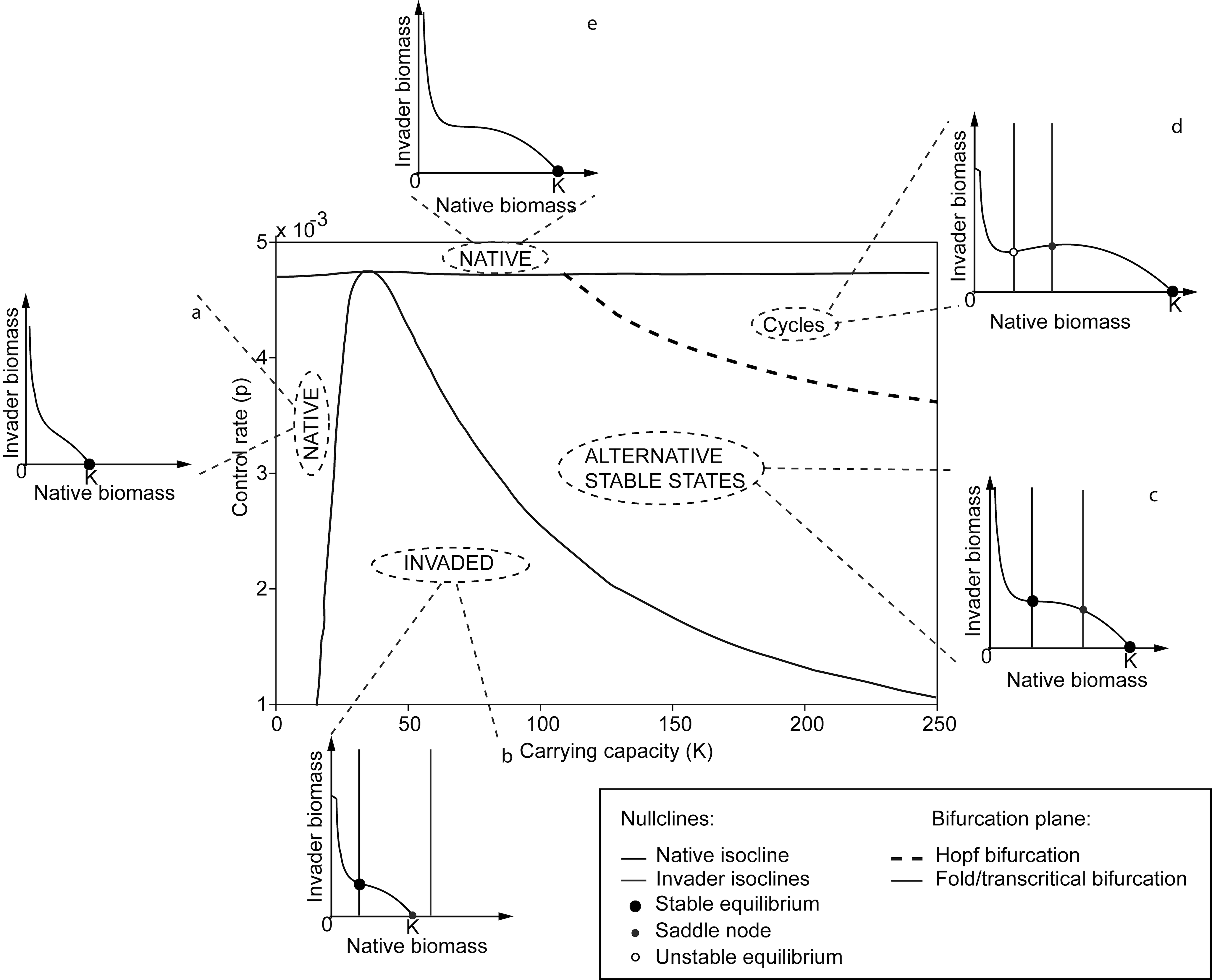


Figure S2: Parameter space illustrating how model dynamics occur under different parameter conditions. In a) there is one stable equilibrium, representing the native-only state; b) the only stable state is invaded, the native equilibrium is a saddle-node. c) With increasing *K* and *p* the native nullcline stretches to the right and the invader ones move left and closer to each other, here the system can exist in either the native-only or invader-dominated state; d) the invaded equilibrium is unstable. e) invader mortality is too high, the only possible equilibrium represents a native-only state.
